# Editorial: Towards control of the HIV epidemic: trends in epidemiology and emerging drug resistance in the integrase inhibitor era

**DOI:** 10.3389/fepid.2026.1991472

**Published:** 2026-10-12

**Authors:** Diego Ripamonti, Maria Santoro, Joseph Fokam, Francesca Ceccherini Silberstein, Josep M. Llibre

**Affiliations:** 1 Department of Medicine, Papa Giovanni XXIII Hospital, Bergamo, Italy; 2 Department of Experimental Medicine, University of Rome Tor Vergata, Rome, Italy; 3 CIRCB, Yaoundé, Cameroon; 4 CIBER Epidemiology & Public Health (CIBERESP), Badalona, Spain

**Keywords:** HIV drug resistance, HIV epidemiology, low-level viremia, molecular network, transmission risk

## Introduction

Over the last 2 decades, the huge advance in HIV pharmacology has contributed to a better control of HIV pandemic with simpler, less toxic and more effective regimens, reducing the number of deaths, new infections and risk of transmission. Despite some countries have achieved the UNAIDS target (1) of 95-95-95% of diagnosed, treated and virologically controlled people with HIV (PWH), most regions still have less than 75% of subjects on treatment having virological control (2). In addition, the extensive roll over to dolutegravir (DTG) in Africa has been followed by treatment emergent resistance to DTG, largely for the high prevalence of reverse transcriptase-associated mutations in these areas (3). This Research Topic expands on the HIV molecular epidemiology, the risk of transmission, the level of pretreatment resistance, the cost of virological failure, the lack of newer classes of ARVs in developing countries in real life, in the integrase inhibitor (INSTI) era and worldwide. Seventeen manuscripts, primarily original researches, are featured, highlighting how much work remains to be done in the aim to achieve the ambitious 95 UNAIDS targets. Here below, the papers are briefly outlined.

### Molecular epidemiology, transmission risk and contact tracing

Gan et al. report on the use of molecular network technology, based on the HIV pol sequence, in order to reveal the transmission links among individuals with HIV infection from MSM population, in Northeast China. They combined degree centrality and betweenness centrality in 1,162 subjects to help identifying individuals at high risk of HIV transmission. This may help informing targeted intervention strategies, as undiagnosed PWH are the primary drivers of sustained HIV transmission (4).

Kirpich et al. report on first systematic molecular epidemiology study of the HIV epidemic in Belarus, in Eastern Europe, revealing two distinct epidemic waves spanning 1997–2005 and 2009–2018, each driven by different dominant modes of transmission. The reconstruction of HIV introduction routes revealed linkages with neighboring Russia and Ukraine, as expected.

Zsichla et al. report on molecular epidemiological analysis of the HIV-1 epidemic in Hungary, analazying partial pol sequences obtained from 1,120 subjects, between 2008 and 2024. Recent infections originate mainly from domestic transmission among men who have sex with men (MSM), with increasing introductions of non-B subtypes from Eastern Europe.

He et al. report a cross-sectional survey in 2022 among 35,513 PWH in Zhejiang province, China, on the risk of sexual transmission based on 4 plasma HIV RNA categories, with minimum risk from having VL <50 copies/mL on ART to high risk being viremic off ART. The vast majority of PWH (88.6%) showed an effective virological control on therapy, with no risk of HIV transmission. Those aged 16–24 years, male, heterosexual and with shorter follow up had increased odds of being at risk of transmission highlighting the need for more focused education in prevention campaign.

Shi et al. report on contact tracing implementation among newly diagnosed PWH in Yunnan Province, China. A total of 1,612 individuals received tracing counseling, revealing 2,226 close contacts, of whom 17.8% (162/910) testing positive for HIV, mostly newly diagnosed. The detection rates for both positive and newly diagnosed infections were higher among contacts identified by recently infected index cases, suggesting how to enhance and refine the efficiency of contact tracing efforts.

### ART efficacy and preventive strategy

Modern ARV- regimens have largely increased the rate of virological suppression in PWH, although majority of people having undetectable HIV RNA. Community viral load expresses the mean of individual plasma HIV RNA levels in a geographical area and it measures the effectiveness of the HIV care cascade along with the potential of transmission (5). Arsuffi et al. report on retrospective longitudinal single-cohort study in northern Italy, confirming the improvement of virological control in the INSTI era, along with changes in the population included in the top 10% of viral loads over time (namely female and non-Italian individuals). This may help identifying subgroups with greater transmission potential and deserving a more intensive monitoring.

Despite being major drivers of incident infections, adolescent girls and young women in Africa continue to have low oral pre-exposure prophylaxis (PrEP) uptake, adherence and persistence (6). Mayanja et al. report on experiences of peer education for oral PrEP use in this population at high risk of HIV acquisition in Uganda, implementing a randomized clinical trial (a qualitative study) with a control group receiving health worker counselling, while the intervention group receiving health worker counselling plus monthly group peer education sessions for four consecutive months. They confirmed the negative impact of community perceptions on PrEP use as major social and cultural barriers to HIV prevention in women.

### HIV drug resistance, virological failure and single tablet regimens

Monitoring transmitted drug resistance (TDR) is necessary to assess the most appropriate regimens in different scenarios. The prevalence of 4-year TDR rate in Nanjing, China (7), was reported to be 8.2%, being higher for NNRTIs (6.7%) than for NRTIs (2.5%) or protease inhibitors (0.1%). Lv et al. report on the high prevalence of V179D/E mutation (14.8%), a polymorphic NNRTI-associated mutation which confers potential low levels of resistance, and its impact on ARV outcomes among ART-naïve PWH in Guangzhou. According to their findings, comparing people with pre-treatment resistance to NNRTI, unique presence of V179D/E and no-resistance mutation group, this mutation may impair early virological response (within the first year). These findings confirm the persistent high frequencies of NNRTI-mutations, the need for genotype testings and the potential role of V179D/E in causing virological failure.

Ouro-Medeli et al. report on TDR in 316 naive subjects in 2023, in Togo, using next-generation sequencing. Although resitance to first generation INSTIs were found in 17.2% of samples, no resistance mutations to dolutegravir (DTG) or to PI were detected. Resistance mutations were detected in 0.6% and 6.3% for NRTIs and NNRTIs, respectively. Their findings confirm the low level of resistance to DTG and support the systematic ongoing switch in that region, notwithstanding the current estimated resistance rate to DTG-based ART in Sub-Saharan Africa (SSA) ranges from 3.9% to 19.6%, in recent studies (3, 8).

Despite the significant scale-up of antiretrovirals (ARVs) in SSA over the last decade, with optimal coverage in some countries (9), the rate of HIV RNA suppression remains alarmingly low in some areas, with a high rate of virological failure. Mac Asaga et al. report on a cross-sectional multicenter cohort of 412 subjects in tertiary care settings, in Nigeria, mostly on NNRTI-based regimes. Along with a high burden of advanced HIV disease (38% with CD4 count <200 cells/mL), only 26.9% and 10.4% had viral load <1000 copies/mL or <40 copies/mL, respectively, with 86% of viraemic subjects harbouring drug resistant HIV-1. Although such tertiary care settings concentrate individuals with resistant HIV and/or adherence issues, these findings confirm the need for genotyping testings at failure and need for newer drugs in real life, if we want to achieve the 95 UNAIDS targets.

Zewdu et al. report on treatment efficacy in 359 young children and adolescents, in a hospital-based retrospective study in Ethiopia, since the introduction of DTG-based regimens in 2019. VF was defined by 1,000 copies/mL, confirmed by two consecutive measurements. Children below 10 years of age were at higher risk of failure, particularly those without parents and with opportunistic infections, confirming the young age as one of the predictors of virological failure in African setting (10).

Persistent Low-level viremia (LLV), despite the inconsistent definitions, may be a clinical dilemma for HIV clinicians with regard to its interpretation, risk of transmission, potential for VF, and its effective management (11). Zheng et al. report on in 9,649 PWH, treated with INSTI-sparing regimens and exhibiting LLV in Guizhou Province (China), from 2016 to 2022. They analyze the impact on virological failure (defined as >1,000 copies/mL after ART), whose rates ranged from 6.1 to 13.7%, according to the LLV thresholds. This study highlighted the need for intensified detection and monitoring efforts particularly among middle-aged and older adults.

In the era of single tablet regimens (STR), simplification to coformulated dual or triple regimens have been rapidly expanding, reducing the risk of drug interactions and potential toxicity and enhancing adherence while preserving the virological and immunological efficacy (12). Mengato et al. report on a multicenter, retrospective observational study (MOSAICO) in 490 PWH on stable therapy in 20 Italian centers. They switched their ART to STR vs. multi-tablet regimens (MTR), between 2017 and 2019. At week 48, real-world effectiveness has confirmed the durability of STR, with INSTI-based regimens being predominant in dual therapy regimens.

### Inequalities in access to care and funding crisis

Although Africa accounts for more than two-thirds of the global burden of HIV/AIDS, few ARV options are available in these countries and, despite the extensive roll over to DTG-based regimens, the rate of virological suppression is still far below the 95% UNAIDS targets in many areas. Emergent DTG resistance and recent funding crisis has further worsend the program support and management (13).

Appiedu-Addo et al. review the persistent gaps and missed opportunities to transform HIV/AIDS into a manageble chronic disease in developing regions as it is in western countries. The double standard is clearly evident in terms of HIV RNA thresholds for virological suppression (<1,000 by WHO *versus* <50 copies/mL in western countries), the standard of care and the access to new classes of antivirals for both treatment and prophylaxis, including long acting options. As long as such gaps are pending, the 95-95-95 UNAIDS targets are far to be achieved in 2030.

### Beyond the undectectability in chronic HIV infection

The metabolic dysfunction associated steatotic liver disease (MASLD) has become a leading cause of chronic liver disease in PWH (14) in the last decade. Hu et al. report on a retrospective cohort study in 814 PWH on stable ART, between 2018 and 2025, at a tertiary care center in China, exploring the predictors of metabolic dysfunction-associated fatty liver disease (14% of them over a median follow-up of 2.8 years), largely driven by the lymphocyte-to-high-density lipoprotein cholesterol ratio, rather than systemic inflammation markers.

While syphilis continues to occur worldwide with 5.36 million new syphilis cases globally in 2021, recent findings confirm the low syphilis testing rates, the high TPPA seroprevalence and association with adverse birth outcomes in HIV population in Uganda (15). Huang et al. report on global burden and trends of syphilis among women of childbearing age (aged 15–49) from 1990 to 2021, based on the information gathered from the Global Burden of Disease (GBD) 2021 database, with burden remaining significant in low-and middle-income countries. As syphilis in pregnancy remains associated with preventable maternal and child adverse events, efforts should focus on this persistent infection in PWH.

## Conclusions

If we want to stop the HIV/AIDS epidemic in areas where the burden of diseases is higher, focused interventions should include more intensive virological monitoring (at least in some subgroups), early genotyping testings, extended availability of more modern classess of antiretrovirals and long term options for both therapy and prophylaxis.
